# Pediatric Emergency Medicine Didactics and Simulation (PEMDAS): Armed Penetrating Trauma

**DOI:** 10.7759/cureus.86017

**Published:** 2025-06-14

**Authors:** Amelia F Wong, Anita Thomas, Jean I Pearce, Elizabeth Sanseau, Michael Levas, Anita Bharath, Cecilia Monteilh, Harold N Lovvorn, Roshan D'Cruz, Rebecca Wilbur, Kimberly C MacKeil-White, Daisy Ciener

**Affiliations:** 1 Pediatric Emergency Medicine, Vanderbilt University Medical Center, Nashville, USA; 2 Pediatrics, Division of Emergency Medicine, University of Pennsylvania School of Medicine and Children’s Hospital of Philadelphia, Philadelphia, USA; 3 Pediatric Emergency Medicine, Medical College of Wisconsin, Milwaukee, USA; 4 Pediatric Emergency Medicine, Children's Hospital of Philadelphia, Philadelphia, USA; 5 Pediatric Emergency Medicine, University of Arizona College of Medicine, Phoenix, USA; 6 Emergency Medicine, Phoenix Childrens Hospital, Phoenix, USA; 7 Pediatric Surgery, Vanderbilt University Medical Center, Nashville, USA; 8 Research, Seattle Children's Hospital, Seattle, USA; 9 Pediatrics, Vanderbilt University Medical Center, Nashville, USA

**Keywords:** airway intubation, chest decompression, concealed weapon, firearm safety, intubation, pediatric emergency medicine, penetrating trauma, trauma resuscitation

## Abstract

Firearms are the leading cause of pediatric death in the U.S., with a rising number of firearm-related injuries presenting to Pediatric Emergency Departments (PED). Trauma centers that manage firearm injuries have higher rates of weapon confiscation. Many healthcare workers have encountered concealed firearms in the ED, despite a lack of workplace firearm handling training. To address this gap, this technical report describes a novel simulation-based curriculum for Pediatric Emergency Medicine (PEM) trainees involving a 15-year-old male presenting with a gunshot wound (GSW) to the chest requiring chest decompression, intubation, and blood products. A concealed firearm discovered on the patient required proper handling protocols. Afterward, a debrief was conducted, and participants completed an evaluation based on pre- and post-simulation knowledge using a 5-point Likert scale (1 = Strongly Disagree, 5 = Strongly Agree). Learners rated the simulation as highly relevant (Mean (M) = 4.8) and realistic (M = 4.7) and reported improvements in teamwork and communication skills (M = 4.6). They also demonstrated increased comfort with identifying firearm injury patterns (pre M = 4.2, post M = 4.6), stabilizing critically ill trauma patients (pre M = 4.1, post M = 4.4), handling firearms in a healthcare setting (pre M = 3.4, post M = 4.3), and functioning effectively as a team in the trauma bay (pre M = 4.5, post M = 4.6). This simulation was well received and provided a psychologically safe environment for trainees to address key knowledge gaps and improve comfort with firearm-related trauma care and safety practices in the pediatric emergency department.

## Introduction

Firearms are the leading cause of pediatric death in the United States. In 2020, there were 10,197 firearm-related deaths (fatality rate 9.91 per 100,000 youth 0-24 years old). Pediatric Emergency Departments (PED) are facing more gun-related visits [1,2]. As a result, there is a critical need for pediatric emergency medicine (PEM) training focused on the management of firearm-related injuries to ensure that healthcare providers are equipped with the knowledge and skills necessary to respond safely and effectively to these life-threatening situations.

Management of firearm injuries involves a systematic and rapid approach to determine scene safety, then stabilize the patient, address life-threatening injuries, and prevent further complications. A primary survey assessing airway, breathing, and circulation should be conducted. Exposing the patient early to identify life-threatening injuries with a penetrating survey should be performed. Afterwards, a thorough secondary survey should be completed to assess other injuries. A thorough history, necessary imaging and labs, and continuous monitoring should be obtained, followed by a safe transition to definitive care.

Trauma centers that manage firearm injuries have higher rates of weapon confiscation [3]. A 2020 American College of Emergency Physicians article reported that 59.1% of American ED health care professionals encounter firearms in or near the ED at least once per year [4]. As a result, metal detectors in PED are increasing [5]. Despite this, a survey of 243 ED physicians revealed 60.7% of physicians had never handled a firearm, and 42.3% reported no firearm safety training [6].

Principles of firearm safety include assumption that the firearm is loaded, keeping the muzzle pointed at the floor away from feet and other people, minimizing handling of the firearm, never trying to unload the firearm, being familiar with hospital protocols, and calling law enforcement to place firearm in a secured area for proper documentation and legal procedure [7].

The subject matter of gunshot wounds (GSW) and firearm handling can be emotionally triggering due to the violence and trauma associated with these scenarios. A simulation-based curriculum provides a psychologically safe environment where trainees can be exposed to these challenging situations without the physical risks of a real-world encounter. Using a high-fidelity manikin, trainees can engage in hands-on, realistic exercises that develop their skills in patient assessment, communication, emergent interventions, and firearm safety protocols. Simulation-based curricula have been developed for firearm handling and penetrating trauma independently; however, to date, no studies have examined the integration of safe firearm handling in the setting a pediatric trauma [8,9].

## Technical report

Development

This simulation case was developed by PEM physicians after a local school shooting. A firearm safety course was taken by the first author. A PEM physician and a Trauma Surgeon with expertise in firearms were consulted. The case was designed to be utilized across the PEM didactics and simulation (PEMDAS) network for PEM fellows, but does benefit from interdisciplinary teams including attendings, residents, medical students, nurses, advanced practice providers, pharmacists, respiratory therapists, and security/safety personnel. Participants had prior experience and knowledge on the management of penetrating trauma; for example, all had taken an Advanced Trauma Life Support (ATLS) course. A link to a free, optional, online firearm handling course was provided in the optional learner preparation in the facilitator guide (Table 1).

**Table 1:** Facilitator Guide PED = Pediatric Emergency Department, PPE = Personal Protective Equipment, GSW = Gun Shot Wound, PICU = Pediatric Intensive Care Unit, IV = Intravenous, EKG = electrocardiogram, ETT = Endotracheal Tube, FAST = Focused Assessment with Sonography for Trauma, CT = Computed Tomography.

Item	Description
Brief narrative description of the case	The patient is a 15-year-old male who presents to the PED with a GSW to the right side of his chest brought by private vehicle. Upon arrival, he appears to be in severe distress. The primary survey will be remarkable for a GSW on the right mid-chest region, deviated airway, decreased breath sounds on the right, and a handgun concealed in the back waistband of his pants. Anticipated early interventions include chest decompression and intubation. The firearm is identified during the evaluation of the patient, and the team alerts the room, notifies law enforcement, and safely secures the firearm. Throughout the case, participants will need to frequently reassess the patient to determine the effectiveness of their interventions. They will conclude with a handover to the PICU or a call to the nearest trauma center.
Participant Roles (required)	Simulation facilitator, team lead, head of the bed, bedside provider
Participant Roles (optional)	Co-simulation facilitator (to act as law enforcement or hospital security, receiving PICU or trauma), bedside nurse, pharmacist, simulation technician, observers
Equipment	High fidelity manikin in street clothes with firearm in the back waist band, Fake Firearm (Please coordinate with your department leadership and security about which fake firearm to use and the timing it will be used), Pediatric Resuscitation Medication references (ex. Broselow tape, PALS card), Monitors, Oxygen, suction, Code Cart with intubation supplies, medications, PPE, IV supplies, Needle and angiocath, Chest tube procedure kit, Chest tube trainers (optional)
Learner Preparation (optional)	1. Optional firearm safety handling course: https://www.onlinetexasltc.com/product/firearm-safety-101-free/
Learning Objectives	1. Prioritize interventions for a critically unstable trauma patient. 2. Identify and manage potential complications associated with penetrating trauma wounds. 3. Execute emergency response to safely secure a firearm found on a patient. 4. Demonstrate effective team leadership, roles, and communication.
Critical Actions #1: Chest Decompression – Finger thoracostomy	Gather equipment: Necessary PPE, Disposable Scalpel, Povidone-Iodine Swabs, Sterile gauze, Hemostatic Forceps, Consider appropriate analgesia (lidocaine). Perform “Finger Thoracostomy” by verbalizing the steps: 1) With the patient supine, place the patient's arm behind their head. 2) Identify the skin incision site between the midaxillary and anterior axillary lines over a rib in the 4th or 5th intercostal space. 3) Clean a wide area of the chest wall area with a Povidone-Iodine swab. 4) Administer local anesthetic solution into the intercostal muscle superior to the expected initial incision. 5) Use the scalpel to make a horizontal skin incision the width of a finger. 6) Use the hemostatic forceps to bluntly dissect a tract into the intercostal space. 7) Anchoring the forceps with your index finger enter the pleural space. 8) Insert a gloved finger into the space and perform a finger sweep to ensure access to the pleural space. 9) Assess for the release of air and/or blood.
Critical Actions #1: Chest Decompression – Needle Decompression	Gather equipment: 5 cm 14-to-16-gauge IV needle/catheter and a 5- or 10-mL syringe. “Needle Decompress” by verbalizing steps: 1) Insert needle catheter attached to syringe along the superior margin of 2nd or 3rd rib in midclavicular or 4th/5th rib space along the anterior axillary line. 2) Advance the needle until the air is aspirated into the syringe. 3) Withdraw needle. 4) Leave Angiocath open to air. 5) Immediate rush of air out of chest. 6) Follow immediately with a standard thoracostomy tube.
Critical Actions #1: Chest Decompression – Chest tube	Gather equipment: Sterile chest tube kit, suture materials, dressing supplies, sterile gloves, scalpel, hemostats, connecting tubing, and drainage system. “Place” chest tube by voicing steps and taping the chest tube onto the mannequin: 1) Position the patient with the right arm raised. 2) Clean and sterilize the insertion site (right 4th or 5th intercostal space in the midaxillary line). 3)Administer lidocaine along the planned path of the chest tube. 4) Make a small incision and use hemostats or fingers to enter pleural space. 5) Insert the chest tube. 6) Connect the chest tube to the drainage system. 7) Suture the chest with sutures. 8) Apply 3-sided occlusive dressing around the insertion site. Chest X-ray Confirmation.
Critical Actions #2: Intubation	Gather intubation equipment: blade, suction, endotracheal tube with backup. Select Rapid Sequence Intubation medication. Pre-oxygenate. If applicable, run through an institutional pre-intubation checklist (Medication plan, Airway plan, backup plan, patient positioning, pre-oxygenation, pulse oximeter on a different extremity than BP cuff, working IV, suction). Intubate. Chest X-ray Confirmation
Critical Actions #3: Identifying and securing the firearm.	Identify the firearm. Notify the charge nurse and contact law enforcement (no law enforcement will be readily available to participants). Clear non-essential personnel and clear the area of bystanders. Secure by the grip, fingers outside of the trigger guard, keeping the muzzle pointed at the floor away from feet and others. Assume the gun is loaded; never try to unload. Law enforcement arrives, and the team hands off the firearm to be placed in a “double” secure area.
Ideal Scenario Flow	Participants were introduced to the scenario of being emotionally charged and dealing with gun violence. A link to a free, optional firearm handling course can be provided. Manikin capabilities are reviewed (can intubate, but no holes shall be placed on the manikin, optional moulage, etc.), and participants are reminded to speak steps out loud and ask for details/equipment they may need. They are given a brief scenario and allotted 2 minutes to assign roles. Participants enter the room to find a patient in obvious distress after sustaining a right chest GSW. They immediately start the primary survey while placing the patient on monitors and working on vascular access. Upon primary survey, they noted the patient was in respiratory distress with a deviated trachea, tachypnea, unilateral breath sounds, tachycardia, and impending shock. The team will provide oxygen, but the patient will remain in respiratory distress with a SpO2 in the 80s. The team will work to decompress the chest and intubate the patient. Respiratory status will improve, but the lung sounds will remain unilateral with hypoxia concerning for a hemopneumothorax. The team will place a chest tube with a 3-sided occlusive dressing, improving vital signs. The team will continue to expose the patient to perform a penetrating survey to find a firearm located in the patient’s back pocket. The team will work to secure the firearm by recognizing the danger of the situation, notifying the charge nurse and law enforcement, and clearing non-essential personnel and bystanders. With no law enforcement readily available, they will keep the patient calm and secure the gun by the grip, keeping the muzzle pointed at the floor away from feet and others while assuming the gun is loaded, and hand the firearm off to law enforcement to be placed in a secure area. The team may order X-rays at this point. If so, films revealing ETT and chest tube in place, with resolving hemopneumothorax and pneumothorax, will be provided. The patient will remain tachycardic, and isotonic fluids/blood products should be ordered, and pain medicines considered. If a FAST is obtained, it will show a positive spine sign in the right upper quadrant view. A Secondary survey and initial diagnostic testing will be ordered, including laboratory studies, X-rays (if not already obtained), EKG, and CT exam. Studies have returned remarkable for lactic acidosis, resolving hemopneumothorax and pneumothorax, normal sinus rhythm, and a pending CT exam. The team continues to monitor the patient's examination closely until the facilitator arrives as the PICU or the nearest trauma center physician calls and asks the team leader for a clinical summary statement.
Anticipated Management Mistakes	1. Failure to recognize impending airway compromise. Most participants will quickly identify the need for chest decompression and intubation given respiratory distress and hypoxia. If participants do not recognize this, the nurse may prompt them by asking them if the airway appears to be deviated. 2. Failure to recognize hemopneumothorax. If participants fail to recognize the need for a chest tube, have the patient remain hypoxic with unilateral lung sounds and progress to obstructive shock. 3. Failure to recognize and safely remove the firearm. Learners should be able to recognize firearms during the initial examination when removing clothing or after rolling patients, however, if there is delay or any confusion, the nurse may draw participants to the weapon by asking if there are any other GSWs or have the team discover it when pelvic X-ray reveals firearm in the pocket.

Equipment/environment

The simulation case utilized a high-fidelity manikin and was conducted in a simulation lab designed to replicate a PED or in an actual PED. Appropriate equipment, simulated medications, and personal protective equipment were provided (Table 1). This list included a link to purchase a fake firearm for the simulation, with instructions to coordinate the timing of the simulation with hospital leadership and security to avoid misunderstandings. One of the critical actions was to decompress the chest and access the pleural space with finger thoracostomy, chest tube, or needle decompression. Participants voiced the steps of the procedure out loud to perform the critical action, but sites had the option to provide procedural kits and task trainers for practice.

Personnel

These simulations were designed to accommodate three to seven learners playing targeted medical personnel working in a PED. A minimum of one simulation instructor was needed to run the simulation, debrief, and act as law enforcement, Trauma Physician, and Pediatric Intensive Care Unit (PICU) attending as needed.

Implementation

Participants were given a pre-brief prior to the simulation, which included specific instructions that the scenario would be emotionally intense and involve gun violence, and were given the option to timeout or step away. The capabilities of the manikin used in the simulation were reviewed, and participants were oriented to the room and supplies available, and who they could call if they wanted help. Participants were reminded to verbalize their actions and request any necessary details or equipment throughout the simulation.

Upon entering the simulation room, participants encountered a 15-year-old male in obvious distress from a GSW to the right chest. The team immediately initiated a primary survey, placed the patient on monitors, started supplemental oxygen, and began establishing vascular access. During the primary survey, the patient was found to be in respiratory distress, exhibiting signs of a hemorrhagic and obstructive shock, including tracheal deviation, tachypnea, unilateral absent breath sounds, and tachycardia with oxygen saturation in the 80s (Table 2).

**Table 2:** Initial Presentation Initial Presentation: HPI = History of Present Illness, PED = Pediatric Emergency Department, HR = Heart Rate, BP = Blood Pressure, T = Temperature, RR = Respiratory Rate, SpO2 = Oxygen Saturation, ETCO2 = End Tidal Carbon Dioxide, GSW = Gun Shot Wound, GCS = Glasgow Coma Scale, CR = Capilarry Refill, A = Airway, B = Breathing, C =Circulation, D = Disability, E = Exposure.

Item	Description
HPI	A 15-year-old male was dropped off in front of the PED with a gunshot wound to the right side of his chest. The patient appears to be in severe distress, complaining of chest pain and difficulty breathing, but is still conscious. Further history is unavailable.
Vital Signs	HR: 118, SpO2: 80%, BP 90/60, RR 40, T 37 C
Primary Survey	A: Patient with trachea deviated to the left. B: Tachypnea, and labored breathing with decreased right lung sounds. C: Tachycardic, 2+ peripheral pulses, CR 2-3 seconds. D: GCS 13, He opens his eyes to commands, is confused but conversive, and follows commands. Pupils are 3 mm and reactive. Able to move all four extremities. E: Penetrating Survey: GSW to the front and back of the right chest, firearm discovered in the patient’s back waistband
Secondary Survey	Head: No bleeding, lacerations, bruising, depressions, or irregularities in the skull. Face: Pupils 3 mm and reactive. Normal tympanic membranes bilaterally. No nasal septal hematoma. No injuries to the mouth. Neck: Left-sided tracheal deviation if not already addressed. Chest: penetrating wound to the right anterior chest. Abdomen: Soft, non-tender, non-distended, without bruising. Pelvis: No hip instability. No blood at the urethral meatus. Limbs: No signs of injuries. Moves all four extremities Back: Penetrating wound to the right upper back. No spinal tenderness

The team then proceeded to decompress the chest by finger thoracostomy, chest tube placement, or needle decompression. Hypoxia persisted, requiring intubation and chest tube placement with subsequent stabilization of the patient’s vital signs (Table 3).

**Table 3:** Case Progression A = Airway, B = Breathing, C =Circulation, D = Disability, E = Exposure, IV = Intravenous, HR = Heart Rate, BP = Blood Pressure, T = Temperature, RR = Respiratory Rate, SpO2 = Oxygen Saturation, ETCO2 = End Tidal Carbon Dioxide, GSW = Gun Shot Wound, GCS = Glasgow Coma Scale, CR = Capilarry Refill, FAST = Focused Assessment with Sonography for Trauma, CT = Computed Tomography, CMP = Comprehensive metabolic panel, CBC = Complete blood count, EKG =  electrocardiogram, PICU = Pediatric Intensive Care Unit, ETT = endotracheal tube, CMP = complete metabolic panel, BUN = blood urea nitrogen, ALT = alanine aminotransferase, AST = aspartate aminotransferase.

Intervention/Time Point	Change in Case	Additional Information
The patient is brought to the trauma bay. Participants to evaluate patient/ 0 min	Learners should establish team roles, activate trauma, assess ABCs, apply monitors, and establish two large-bore IVs. Exam as above.	A: Patent, trachea deviated to the left. B: Labored breathing with decreased right lung sounds. C: Tachycardic, 2+ pulses, CR 2-3 sec. D: GCS 13: He opens his eyes to commands, is confused but conversive, and follows commands. Pupils are 3 mm and reactive. Able to move all four extremities. E: Upon exposure / penetrating survey GSW to the front and back of the right chest, the firearm was discovered in the patient’s back waistband.
Respiratory distress/ 1 min	The patient is hypoxic to 80%. Learners should apply oxygen.	HR 120, SpO2 80%, BP 95/60, RR 32, T 37, ETCO2 30. The patient will remain hypoxic, with a deviated trachea, in respiratory distress despite non-invasive respiratory support.
Airway - Chest Decompression	Learners should prepare to decompress the chest. If this is not considered by the team, have the facilitator point out the deviated trachea, hypoxia, and tachycardia. Finger Thoracostomy Option - Gather equipment: Necessary PPE, Disposable Scalpel, Povidone-Iodine Swabs, Sterile gauze, Hemostatic Forceps, Consider appropriate analgesia (lidocaine). Perform “Finger Thoracostomy” by verbalizing the steps: 1) With the patient supine, place the patient's arm behind their head. 2) Identify the skin incision site between the midaxillary and anterior axillary lines over a rib in the 4th or 5th intercostal space. 3) Clean a wide area of the chest wall area with a Povidone-Iodine Swab. 4) Administer local anesthetic solution into the intercostal muscle superior to the expected initial incision. 5) Use the scalpel to make a horizontal skin incision the width of a finger. 6) Use the hemostatic forceps to bluntly dissect a tract into the intercostal space. 7) Anchoring the forceps with your index finger, enter the pleural space. 8) Insert a gloved finger into the space and perform a finger sweep to ensure access to the pleural space. 9) Assess for the release of air and/or blood. Need Decompression Option - Gather equipment: 5 cm 14- to 16-gauge IV needle/catheter and a 5- or 10-mL syringe. “Needle Decompress” by verbalizing steps: 1) Insert needle catheter attached to syringe along the superior margin of the 2nd or 3rd rib in midclavicular or 4th/5th rib space along the anterior axillary line. 2) Advance the needle until the air is aspirated into the syringe. 3) Withdraw the needle. 4) Leave the Angiocath open to air. 5) Immediate rush of air out of the chest. 6) Follow immediately with a standard thoracostomy tube. Oxygenation will improve to 85%; however, lung sounds will still be diminished at the right lower lung base.	HR 112, SpO2 80%, BP 95/60, RR 25, T 37, ETCO2 35
Airway - Intubation	Intubation - Gather intubation equipment: blade, suction, endotracheal tube with backup. Select Rapid Sequence Intubation medication. Pre-oxygenate. If applicable, run through an institutional pre-intubation checklist (Medication plan, Airway plan, backup plan, patient positioning, pre-oxygenation, pulse oximeter on a different extremity than BP cuff, working IV, suction). Intubate. Chest X-ray Confirmation.	HR 112, SpO2 85%, BP 95/60, RR 25, T 37, ETCO2 35
Breathing - Hypoxia and Unilateral breath sounds/ 8 min	Chest Tube Placement - Gather equipment: Sterile chest tube kit, suture materials, dressing supplies, sterile gloves, scalpel, hemostats, connecting tubing, and drainage system. “Place” chest tube by voicing steps and taping the chest tube onto the mannequin: 1) Position the patient with the right arm raised. 2) Clean and sterilize the insertion site (right 4th or 5th intercostal space in the midaxillary line). 3) Administer lidocaine along the planned path of the chest tube. 4) Make a small incision and use hemostats or fingers to enter the pleural space. 5) Insert the chest tube. 6) Connect the chest tube to the drainage system. 7) Suture the chest with sutures. 8) Apply an occlusive dressing around the insertion site. Chest X-ray Confirmation.	HR 105, SpO2 95%, BP 95/60, RR 25, T 37, ETCO2 35. If a chest x-ray is obtained, show participants a chest x-ray with properly placed ETT, and chest tube in the proper position, with resolving hemo/pneumothorax.
Circulation – Tachycardia and delayed capillary refill/ 12 min	Learners continue to examine the patient and should recognize and treat hemorrhagic shock with appropriate IV fluids and blood product resuscitation, and activate the institutional mass transfusion protocol. Tachycardia could also represent pain, and learners should consider pain medicines. If interventions are completed, HR improves to the 90’s.	HR 105, SpO2 95%, BP 95/60, RR 25, T 37, ETCO2 40. A: ETT in place. B: Improved aeration of the right lung base. C: Tachycardic, 2+ pulses, CR 2-3 sec. D: Sedated, Pupils 3 and reactive. E: Upon exposure/penetrating survey GSW to the front and back of the right chest, the firearm is discovered in the patient’s back pocket.
Exposure - Firearm is discovered/ 15 min	Learners should roll the patient to finish the examination and discover the firearm. Upon discovery, learners should: Acknowledge danger and notify the charge nurse and law enforcement (no law enforcement will be readily available to participants until someone from the medical team secures the firearm). Clear non-essential personnel and clear areas of bystanders. Secure by the grip, keeping the muzzle pointed at the floor away from feet and others. All while assuming the gun is loaded and never attempting to unload. Hand the firearm off to law enforcement to be placed in a secure area. If the team does not roll the patient, have the nurse prompt participants, asking, “Are there any other GSWs?”	HR 90, SpO2 95%, BP 120/70, RR 25, T 37, ETCO2 40. A: ETT in place. B: Improved aeration of the right lung base. C: Tachycardic, 2+ pulses, CR 2-3 sec. D: Sedated, Pupils 3 and reactive. E: Upon exposure/penetrating survey GSW to the front and back of the right chest, the firearm is discovered in the patient’s back waistband.
Secondary Survey	Learners should complete a secondary survey	HR 90, SpO2 95%, BP 100/60, RR 25, T 37, ETCO2 40. Head: No bleeding, lacerations, bruising, depressions, or irregularities in the skull. Face: Pupils 3 mm and reactive. Normal tympanic membranes bilaterally. No nasal septal hematoma. No injuries to the mouth. Neck: Trachea now midline. Chest: penetrating wound to the right anterior chest. Abdomen: Soft, non-tender, non-distended, without bruising. Pelvis: No hip instability. No blood at the urethral meatus. Limbs: No signs of injuries. Moves all four extremities. Back: Penetrating wound to the right upper back. No spinal tenderness.
Participants order studies/ 20 min	Participants should consider ordering: FAST exam, blood gas, lactate, CMP, CBC, lipase, type and screen, coagulation studies, EKG, chest X-Ray (if not already ordered), pelvic X-Ray, CT Chest Abdomen Pelvis with contrast. If the participants do not request any labs, can have the nurse prompt, “Do you want me to collect any more blood for labs?”	HR 90, SpO2 98%, BP 100/60, RR 25, T 37, ETCO2 40. FAST exam: (+) Spine sign in right upper quadrant, negative elsewhere. Venous Blood Gas: pH=7.24, pCO2=48, pO2= 75, Bicarbonate = 13. Lactate: 3. CMP: Sodium 144, Potassium 4.7, Chloride 111, CO2 25, BUN 13, Creatinine 0.9, Glucose 132, Calcium total 10.2, ALT 200, AST 216. CBC: White blood cell count: 15, Hematocrit: 30, Hemoglobin: 10, Platelets: 196. EKG: Normal Sinus Rhythm with rate of 90. Chest X-Ray: depending on the interventions, show X-ray +/- ETT tube and Chest tube in place. Pelvic X-Ray: No fractures or dislocations +/- firearm in pocket depending on removal. CT scan(s): pending.
Completion and sign-out to PICU/ 25 min	The team should identify that the patient requires PICU/Trauma admission and give a sign-out to the PICU team. Sample sign-out: “This is a 15-year-old male presenting with GSW to the right chest resulting in tension pneumothorax and hemothorax requiring chest decompression, intubation, and chest tube placement. Studies were remarkable for mixed respiratory and metabolic acidosis. FAST exam (+) spine sign in the right upper quadrant view and negative elsewhere. He has received fluids/blood and remains intubated with serosanguinous output from the chest tube. He has a post-intubation sedation drip running and pain medicines have been given. The CT exam is pending. A firearm was found on his body and secured and now with law enforcement.”	The facilitator can play the role of PICU attending or receiving trauma facility physician.

While gaining exposure, the team discovered a firearm in the patient’s back waistband. This prompted them to immediately address the firearm by either utilizing their existing hospital policy (if applicable), alerting the room, notifying the charge nurse and law enforcement, and by clearing the area of non-essential personnel. Without immediate law enforcement presence, the team secured the firearm safely, treating it as loaded. The firearm was then handed over to law enforcement for placement in a secure area (Table 3).

**Figure 1:**
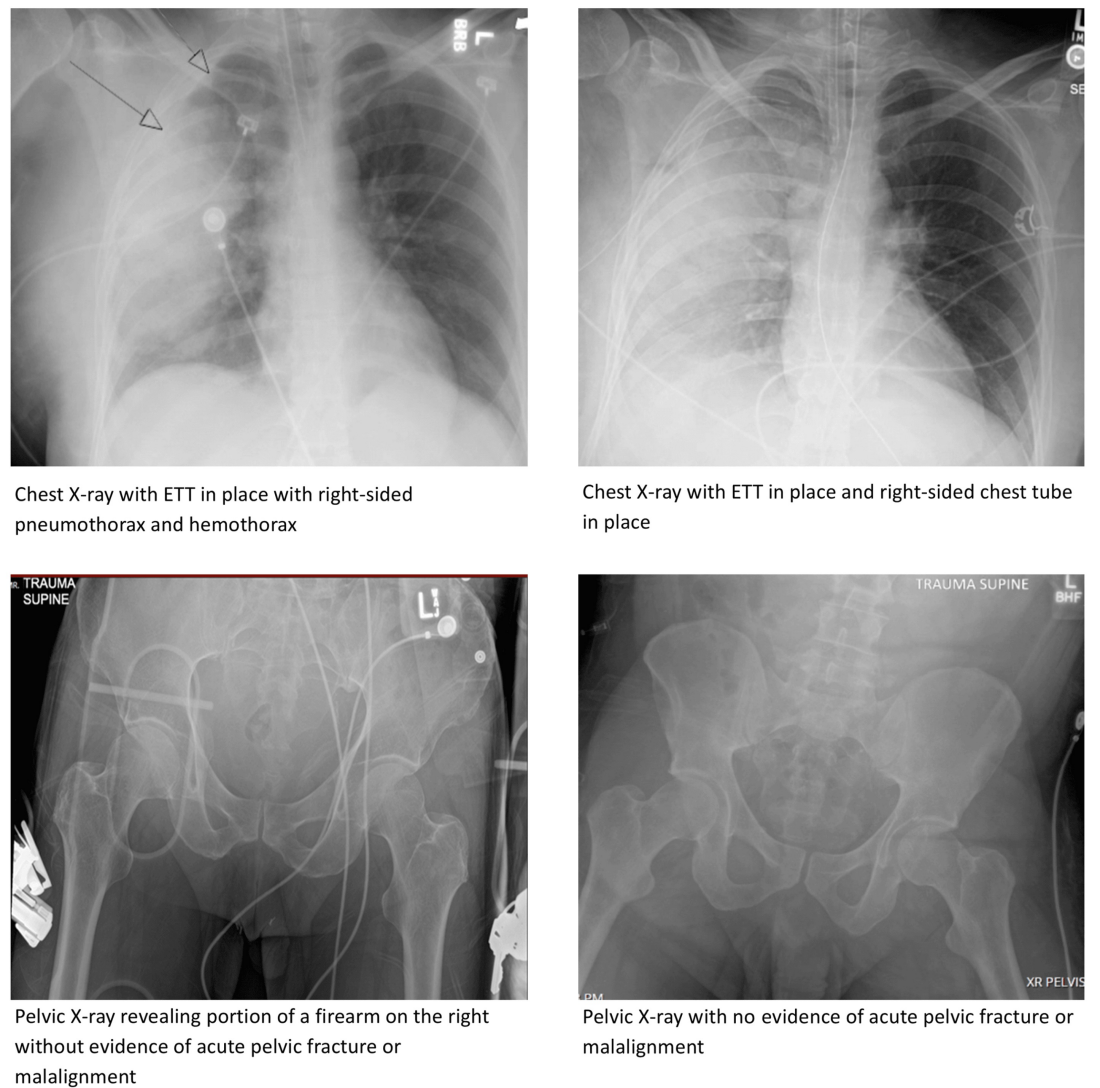
Simulation X-Rays Revealing a Hemopneumothorax and a Normal Pelvis. ETT = Endotracheal Tube Figure Credit: Author.

**Figure 2:**
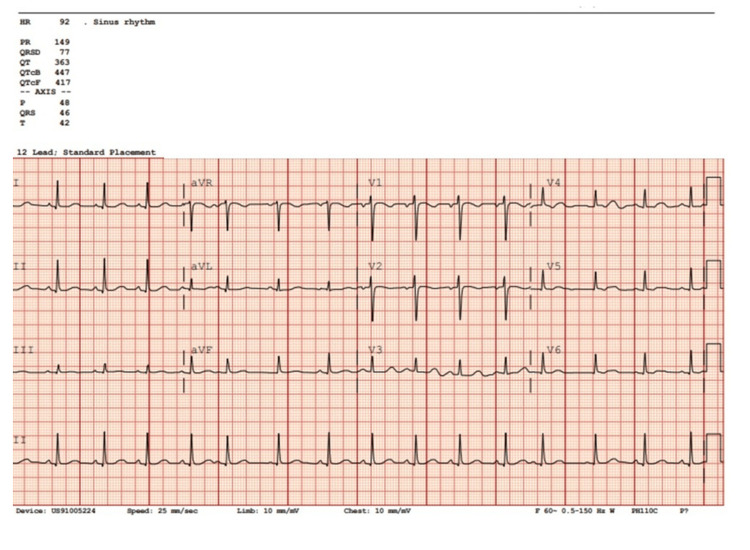
Simulation EKG Revealing Sinus Tachycardia

The team was expected to perform a comprehensive secondary survey and could request initial diagnostic testing, including laboratory studies (Table 4), X-rays (Figure 1), EKG (Figure 2), and a CT scan of the chest, abdomen, and pelvis. A chest X-ray reveals correct placement of the endotracheal tube (ETT) and chest tube, along with resolving hemopneumothorax. Pelvic X-ray showed no evidence of acute pelvic fracture or malalignment. If the team had not detected the concealed firearm prior to imaging, the pelvic X-ray would reveal a firearm in the patient’s waistband. Lab results were consistent with mild anemia and lactic acidosis. An EKG would reveal a normal sinus rhythm. CT scans remained pending.

**Table 4:** Simulation Labs Revealing a Metabolic Acidosis and Anemia VBG = venous blood gas, pCO₂ = partial pressure of carbon dioxide, pO₂ = partial pressure of oxygen, BUN = blood urea nitrogen, ALT = alanine aminotransferase, AST = aspartate aminotransferase, PTT = partial thromboplastin time, PT = prothrombin time, INR = international normalized ratio.

Panel	Test	Result
Venous Blood Gas (VBG)	pH	7.24
	pCO₂	48 mmHg
	pO₂	75 mmHg
	Bicarbonate	13 mEq/L
	Lactate	3 mmol/L
Complete Metabolic Panel	Sodium	144 mEq/L
	Potassium	4.7 mEq/L
	Chloride	111 mEq/L
	CO₂ (serum)	25 mEq/L
	BUN	13 mg/dL
	Creatinine	0.9 mg/dL
	Glucose	132 mg/dL
	Calcium (total)	10.2 mg/dL
	ALT	200 U/L
	AST	216 U/L
Complete Blood Count	White Blood Cells	15 x10⁹/L
	Hematocrit	30%
	Hemoglobin	10 g/dL
	Platelets	196 x10⁹/L
Coagulation Panel	PTT	30 sec
	PT	14.1 sec
	INR	1.0
Type and Screen		Pending

**Figure 3:**
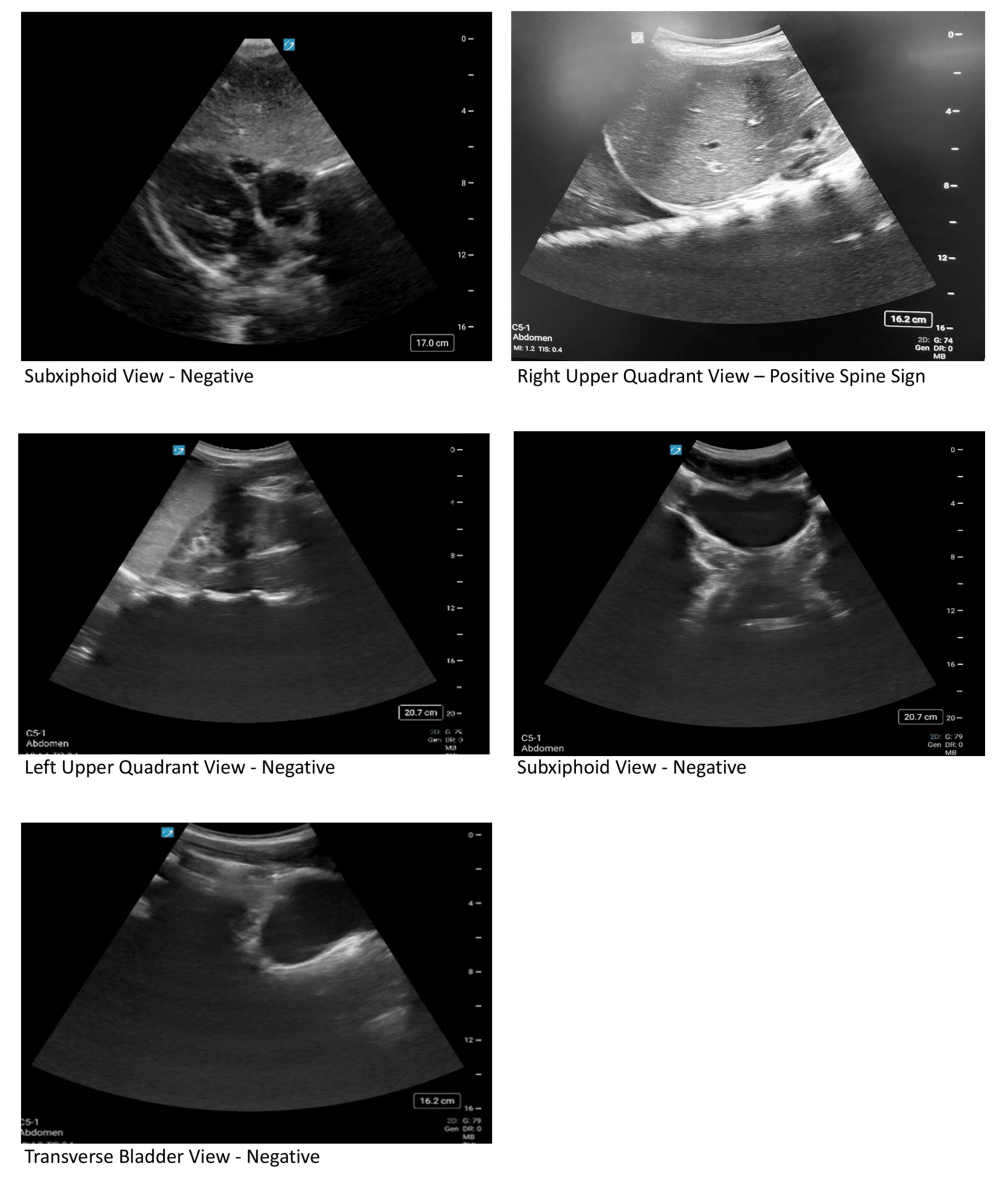
Simulation FAST Exam Revealing a Positive Spine Signs in the Right Upper Quadrant View FAST = Focused Assessment with Sonography for Trauma Figure Credit: Author

The patient remained tachycardic, prompting the team to order isotonic fluids, blood products, and pain management. A FAST (Focused Assessment with Sonography for Trauma) (Figure 3), if obtained, would show fluid at the base of the right lung. The team continued to monitor the patient’s condition until the facilitator assumed the role of an ICU physician or trauma physician and requested a clinical summary from the team leader.

Debriefing

**Figure 4:**
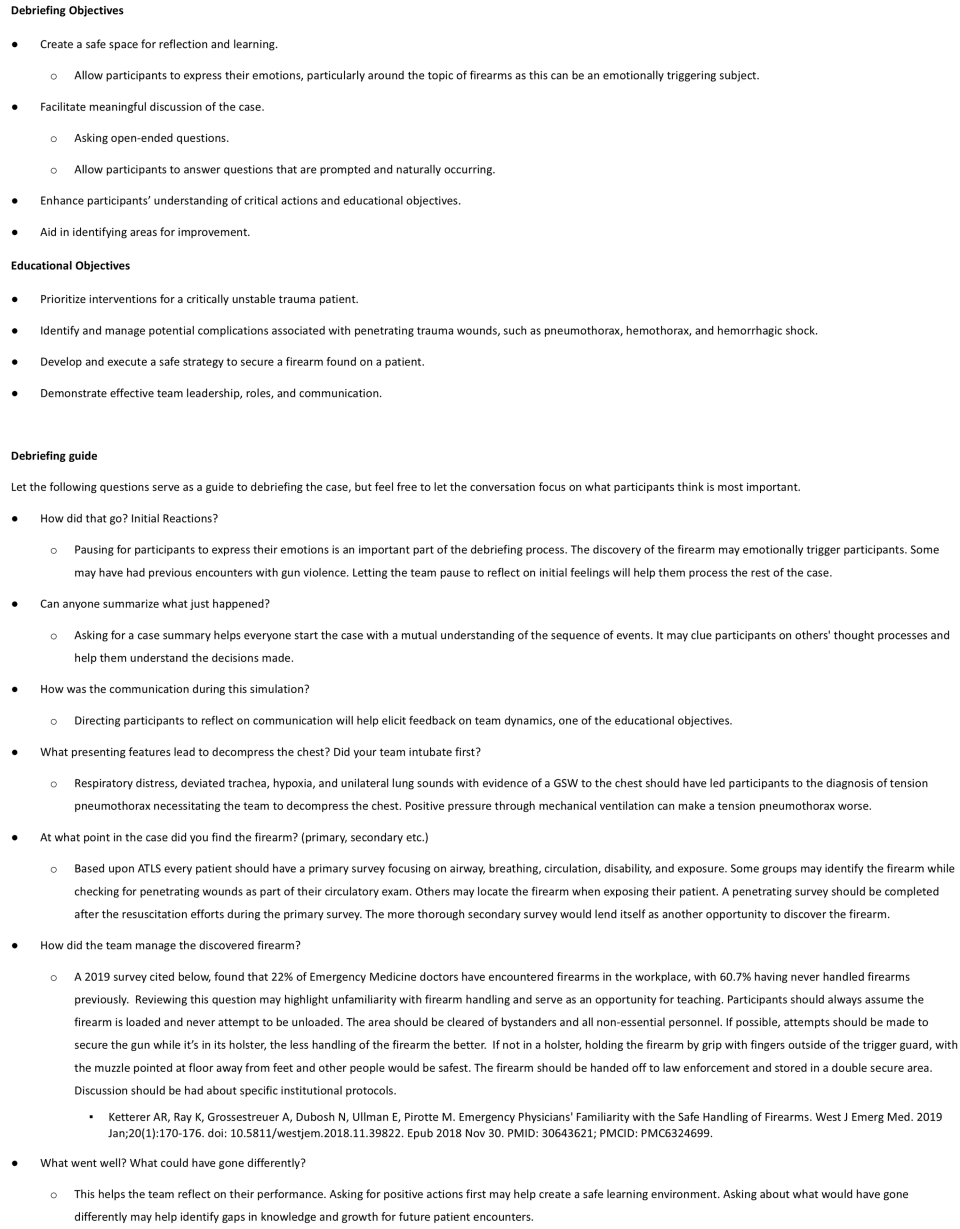
Debriefing Guide This debriefing guide was developed by the author. Permission for use and publication granted by the author.

The debriefing session was conducted using the PEARLS (Promoting Excellence and Reflective Learning in Simulation) debriefing framework [10], supported by additional tools and resources (Figure 4). Facilitators began the session by inviting participants to share their reflections on the scenario, followed by a structured discussion guided by the debriefing outline. This approach allowed the conversation to naturally incorporate elements of crisis resource management skills such as teamwork and communication, while also reinforcing key principles of penetrating trauma management and safe firearm handling.

Assessment

**Figure 5:**
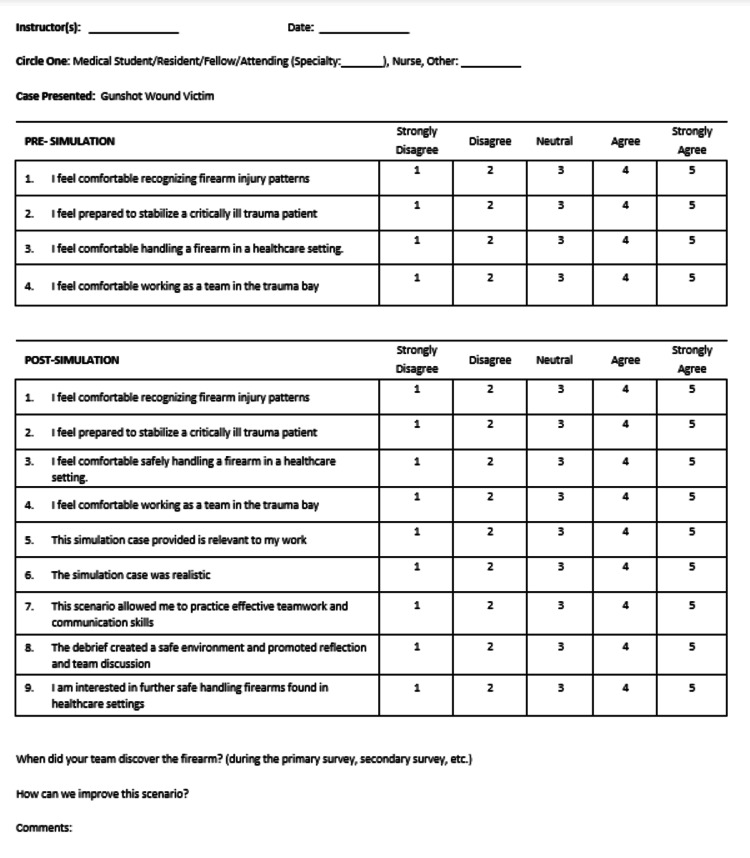
Evaluation Form This evaluation form was created by the author for the purposes of this study. Permission for use and publication granted by the author.

All facilitators running the simulations were either current PEM fellows, faculty, or nursing education specialists, with some faculty having additional simulation-based medical education training or experience. Facilitators provided feedback on participant performance, focusing on both team dynamics and resuscitation and procedural skills. After the debriefing, participants completed an evaluation form (Figure 5) to answer based on the knowledge base both before and after the simulation. The pre-simulation knowledge assessment was done after the simulation, not to give away the case. Questions assessed the relevance, realism, and educational value of the simulation. The feedback was collected using a Likert scale (1 = strongly disagree, 2 = disagree, 3 = neutral, 4 = agree, 5 = strongly agree) and free response questions. The mean (M) and median were recorded for each statement.

Results

The simulation was implemented at four geographically distinct institutions with a total of 59 participants, made up of 29 PEM fellows, 26 emergency medicine residents, two nurses, and two PEM attendings. Immediately after the simulation, a debrief was conducted to elicit participants’ reactions - Level 1 of Kirkpatrick's Four-Level Training Evaluation Model [10,11] - and allow for emotional decompression. Educational objectives were reviewed and gaps in knowledge were addressed in detail, fulfilling Level 2 (Learning) of Kirkpatrick's model. Following the debrief, participants completed the evaluation form (Figure 5) to answer questions based on the knowledge base before and after the simulation.

Overall, the simulation was well received and highly rated for being relevant to their work (M = 4.8, median 5) and realistic (M = 4.7, median 5). The simulation allowed the team to practice effective teamwork and communication skills (M = 4.6, median 5). The debrief created a safe environment and promoted reflection and team discussion (M = 4.8, median 5) (Table 5).

**Table 5:** Participant Cumulative Evaluation Scores (N = 59)

Statement	M	Median
This simulation case provided is relevant to my work	4.8	5
The simulation case was realistic	4.7	5
This scenario allowed me to practice effective teamwork and communication skills	4.6	5
The debrief created a safe environment and promoted reflection and team discussion	4.8	5
I am interested in further safe handling firearm training in the healthcare setting	4.3	5
Rated on a 5-point Likert scale (1 = strongly disagree, 2 = disagree, 3 = neutral, 4 = agree, 5 = strongly agree).		

**Figure 6:**
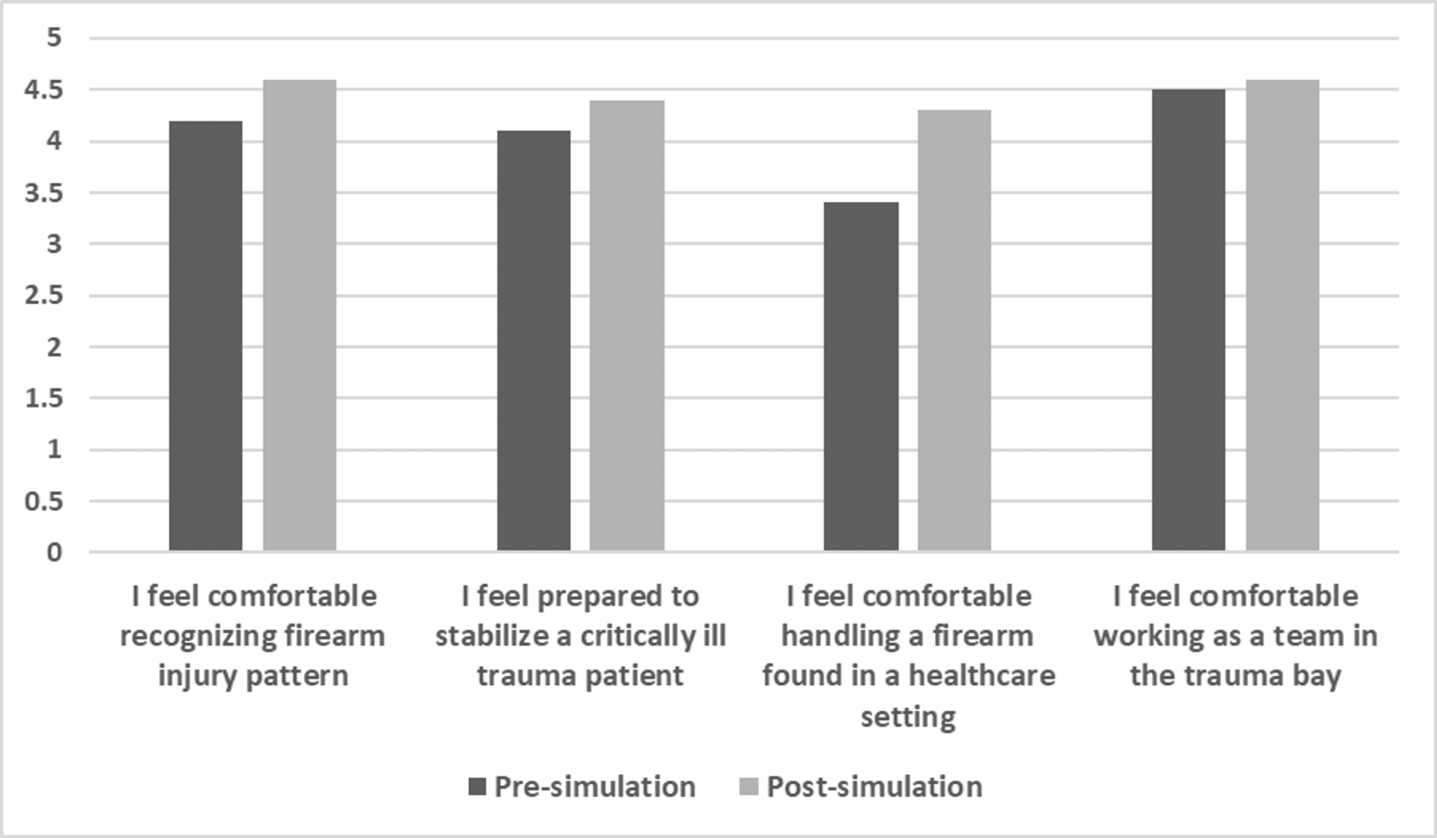
Pre-Simulation and Post-Simulation Evaluation Scores (n = 59) Rated on a 5-Point Likert Scale (1 = Strongly Disagree, 2 = Disagree, 3 = Neutral, 4 = Agree, 5 = Strongly Agree).

The simulation demonstrated effective learning (Level 2 of Kirkpatrick's model). Comparing knowledge base pre-simulation and post-simulation, participants reported improved comfort recognizing firearm injury patterns (pre-simulation M = 4.2, post-simulation M = 4.6), preparedness to stabilize a critically ill trauma patient (pre-simulation M = 4.1, post-simulation M = 4.4), comfort in handling a firearm found in a healthcare setting (pre-simulation M = 3.4, post-simulation M = 4.3), and comfort working as a team in the trauma bay (pre-simulation M = 4.5, post-simulation M = 4.6) (Figure 6). There was variation in the timing of firearm discovery in the case, including the penetrating survey, secondary survey, and some groups never finding the firearm (Table 6). Valuable feedback was provided for simulation improvement (Table 7). Furthermore, the case generated further interest in safe handling firearm training in the healthcare setting (M = 4.3, median 5) (Table 5).

**Table 6:** Themes Obtained From Participant Reponses to “How could we improve this scenario?” FAST = Focused Assessment with Sonography for Trauma

Theme	Representative Quote
No changes	“It was great!”
	“N/A”
	“Loved it.”
Making the simulation more multidisciplinary	“I feel like firearm safety for nurses in the ED would be smart. As there might be a slight delay of law enforcement arriving to the room and a nurse/charge nurse might be standing at the door securely holding a firearm until they arrive.”
	“Law enforcement for additional education!”
	“Having family members present.”
	“Including social work.”
	“Including nurses.”
Adding educational components	“Including a trauma review”
	“Adding a video of FAST exam”
	“Repetition”

**Table 7:** Free Responses to “When did your team discover the firearm?”

Theme	Representative Quote
Primary Survey	“During exposure”
	“Penetrating survey”
Secondary Survey	“Secondary survey”
	“Secondary survey when rolling”
Other	“Late in the game”
	“Never”

## Discussion

This educational innovation aimed to enhance PEM training by developing and implementing a simulation focused on the management of firearm-related injuries and safe firearm handling in the PED setting. The simulation was designed to provide an immersive, hands-on learning experience for PEM fellows and interdisciplinary teams, enabling them to practice critical skills in a controlled environment. Overall, the simulation showed improvement in participants' comfort and preparedness in managing GSWs and handling firearms encountered in the PED, as evidenced by positive feedback and self-reported gains in knowledge and confidence.

The development of this simulation required careful consideration of both the clinical complexities of GSW management and the sensitive nature of firearm handling in the ED. The inclusion of a clearly fake firearm provided a tangible yet psychologically safe learning environment. The use of high-fidelity manikins added to the realism to achieve educational objectives.

One important lesson learned was the variability in the management of penetrating chest trauma with evidence of tension pneumothorax. A tension pneumothorax should be decompressed immediately [12,13]. We consulted with pediatric trauma surgeons who recommended doing so by finger thoracostomy or chest tube placement over needle decompression, given the mechanism of injury with a likely hemothorax component, and the higher failure rates associated with needle decompression [14]. They also recommended preparing to secure the airway simultaneously. Some groups intubated before chest decompression, while others avoided positive pressure until the chest was decompressed. This variability may have stemmed from differences in how the manikin displayed clinical signs or what findings were elicited from the facilitator. Another inconsistency across groups was the timing of firearm discovery during the simulation. This emphasized the importance of consistently incorporating a penetrating trauma survey during the exposure phase of the primary survey. The debriefing sessions proved essential for allowing participants to reflect on their performance, explore their emotional responses, and engage in discussions about firearm-related safety and weapon screening. Many of the debriefings centered around the moment of firearm discovery, underlining the distinct value of this scenario in addressing a challenging yet increasingly relevant aspect of emergency care.

Future iterations of this simulation could benefit from the inclusion of a full trauma team, including nursing, social work, and law enforcement, to further enhance realism and interdisciplinary coordination. Expanding the scenario to allow more time or using task trainers and higher-fidelity manikins capable of supporting procedural tasks such as chest decompression may further enrich the learning experience. Additionally, participant feedback revealed interest in more comprehensive firearm safety education within healthcare settings. This aligns with a broader need for standardized protocols and formal training in firearm handling, which could contribute to improved safety and reduced risk of accidental injury in the clinical environment.

Limitations

Despite its successes, the simulation has several limitations. One significant limitation is the challenge of generalizability, as the simulation was conducted in a controlled environment, mainly with PEM fellows and ED residents with trauma experience. Trauma center designation may play a role in management across different institutions. There were both level 1 trauma centers and non-trauma facilities that participated in this study. There were technical limitations of what the high-fidelity manikins were able to demonstrate, such as signs of tension pneumothorax as mentioned above. Another limitation is the ability to perform chest decompressions on the manikins. The unrealistic firearm could take away from the case's realism but was purposeful to avoid emotional trauma and comply with hospital security measures. Lastly, the evaluation form relied on self-reported data, which may be subject to bias.

## Conclusions

This simulation addressed a critical gap in pediatric emergency medicine training by integrating firearm-related injury management and safe firearm handling into a realistic, team-based learning scenario. This technical report presents a curriculum that was well-received and led to reported improvements in provider preparedness and confidence. As firearm encounters in pediatric settings continue to rise, embedding this type of education into routine training is a necessary step toward improving provider readiness, team coordination, and overall safety in the emergency department.
